# Ultrasound Stimulation of Prefrontal Cortex Improves Lipopolysaccharide-Induced Depressive-Like Behaviors in Mice

**DOI:** 10.3389/fpsyt.2022.864481

**Published:** 2022-04-29

**Authors:** Sha-sha Yi, Jun-jie Zou, Long Meng, Hou-minji Chen, Zhong-qiu Hong, Xiu-fang Liu, Umar Farooq, Mo-xian Chen, Zheng-rong Lin, Wei Zhou, Li-juan Ao, Xi-quan Hu, Li-li Niu

**Affiliations:** ^1^Institute of Biomedical and Health Engineering, Shenzhen Institutes of Advanced Technology, Chinese Academy of Sciences, Shenzhen, China; ^2^School of Rehabilitation, Kunming Medical University, Kunming, China; ^3^Department of Rehabilitation Medicine, The Third Affiliated Hospital, Sun Yat-sen University, Guangzhou, China

**Keywords:** transcranial ultrasound, brain stimulation, depression, inflammatory cytokines, neuromodulation

## Abstract

Increasing evidence indicates that inflammatory responses may influence brain neurochemical pathways, inducing depressive-like behaviors. Ultrasound stimulation (US) is a promising non-invasive treatment for neuropsychiatric diseases. We investigated whether US can suppress inflammation and improve depressive-like behaviors. Mice were intraperitoneally injected with lipopolysaccharide to induce depressive-like behaviors. Ultrasound wave was delivered into the prefrontal cortex (PFC) for 30 min. Depressive- and anxiety-like behaviors were evaluated through the forced swimming test (FST), tail suspension test (TST), and elevated plus maze (EPM). Biochemical analyses were performed to assess the expression of inflammatory cytokines in the PFC and serum. The results indicated that US of the PFC significantly improved depressive-like behaviors in the TST (*p* < 0.05) and FST (*p* < 0.05). Anxiety-like behaviors also improved in the EPM (*p* < 0.05). Furthermore, the lipopolysaccharide-mediated upregulation of IL-6, IL-1β, and TNF-α in the PFC was significantly reduced (*p* < 0.05) by US. In addition, no tissue damage was observed. Overall, US of PFC can effectively improve lipopolysaccharide-induced depressive-like behaviors, possibly through the downregulation of inflammatory cytokines in the PFC. US may be a safe and promising tool for improvement of depression.

## Introduction

Depression is a serious, heterogeneous, and recurrent severe mental illness and is the leading cause of disability and suicide worldwide (1, 2). Currently, there are more than 300 million people experiencing a depressive disorder worldwide (3) with more than 800,000 dying by suicide each year (4). The World Health Organization (WHO) predicts that depression will be the leading cause of disease burden by 2030 (5). In addition, depression is a symptom of several medical conditions, including pain (6), stroke (7), Alzheimer’s disease (8), and Parkinson’s disease (9). Therefore, effective tools to treat depression are urgently needed.

Studies have indicated that structural and functional abnormalities underlie depression (10, 11), particularly in the brain (12, 13). Specifically, histopathological evidence has shown that patients with major depressive disorder present with decreased cortical thickness, neuronal size, and glial density in the prefrontal cortex (PFC) (14) and hippocampus (15, 16). Drevets et al. found that patients with depression displayed reduced brain activity in the ventral PFC (17), and their depressive behavior was closely related to dendritic atrophy and synaptic remodeling in the PFC and hippocampus (18, 19).

In recent years, it has been reported that deep brain stimulation (DBS) targeting the PFC is effective for the treatment of anhedonia, anxiety, and cognition disorder in Wistar rats with treatment-resistant depression (20). Non-invasive transcranial magnetic stimulation (TMS) has been proved to reduce negative emotions and enhance pleasure by improving synaptic plasticity and neuronal metabolism in the PFC (21, 22). In clinical practice, TMS has been shown to have antidepressant effects by changing both global connectivity and local excitability (23). With its extraordinary spatial resolution, optogenetic stimulation of specific neurons in the PFC increases the synaptic number and function and reproduces the actions of antidepressant drugs (24). However, TMS, DBS, and optogenetic stimulation have low penetration depth or are invasive. Therefore, new non-invasive tools are needed that can afford high spatial resolution and deeply penetrate the brain.

Ultrasound is a promising non-invasive neuromodulation technology with the advantage of high spatial resolution and great penetration depth (25). Previous studies have found that low-intensity focused ultrasound (LIFUS) has an impact on the function of ion channels and the firing of neurons (26, 27). In addition, many studies have demonstrated that LIFUS has promising intervention results in animal models of Alzheimer’s disease (28, 29), Parkinson’s disease (30), epilepsy (31, 32), disturbance of consciousness (33–35) and stroke (36, 37). Zhang et al. found that the delivery of ultrasound to the rat prelimbic cortex upregulates hippocampal brain-derived neurotrophic factor (BDNF) and improves depressive-like behaviors (38). Another recent study reported that ultrasound stimulation (US) of the ventromedial PFC of rats subjected to chronic unpredictable stress increased sucrose preference and reduced immobility time in the forced swimming test (FST), and indicated that ultrasound could improve depressive-like behaviors by enhancing the signaling pathways downstream of BDNF in the PFC (39). The roles of the immune system and inflammation in depression have also earned considerable attention (40, 41), and its increased activation may serve as an effective therapeutic target. Specifically, recent studies have shown the potential of ultrasound-mediated neuromodulation to prevent or treat inflammatory diseases (42–44). Therefore, we investigated whether ultrasound targeting of the PFC could non-invasively modulate the immune system and inflammatory response to improve depressive-like behaviors in mice.

## Materials and Methods

### Ethics Statement

The study protocol was approved by the Animal Experimental Ethics Committee of the Shenzhen Institutes of Advanced Technology, Chinese Academy of Sciences. All animal experiments were conducted in accordance with the guidelines of Care and Use of Research Animals established by the Animal Research Committee at Shenzhen Institutes of Advanced Technology, Chinese Academy of Sciences (approval number: SIAT-IRB-150213-YGS-ZHR-A0094-2).

### Animals and Experimental Design

Seven-week-old male C57BJ/6J mice (18–22 g in weight) were purchased from Beijing Vital River Laboratory Animal Technology (BioRiver, Beijing, China). All mice were kept in a room under a 12 h reversed light/dark cycle with stationary temperature (23^°^C–25°C), 50–60% relative humidity, and an adequate supply of water and food.

A preliminary study was conducted to assess the optimal dose of lipopolysaccharide (LPS) for induction of depressive-like behaviors. Mice were injected with 0.5, 0.83, or 1 mg/kg of LPS, or equivalent amounts of 0.9% saline. Behavioral phenotypes were assessed in the open field test (OFT) 24 h after LPS injection. The number of entries in the central zone was recorded.

**FIGURE 1:**
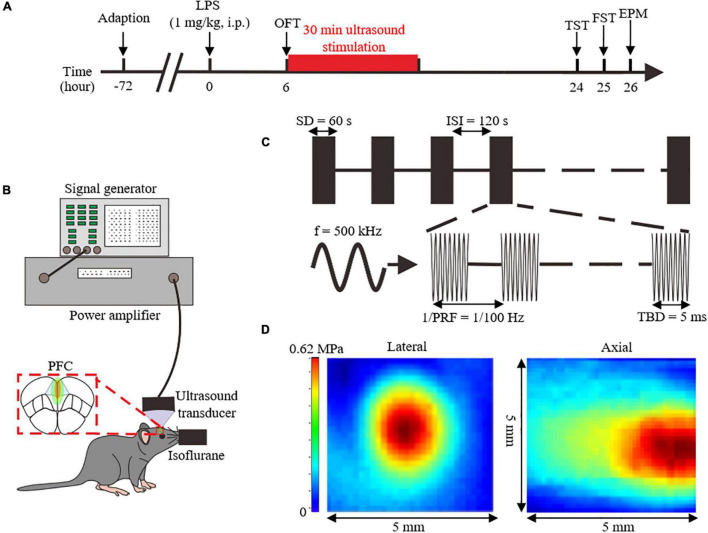
Overview of the experimental design and analyzed parameters. **(A)** Experimental process flowchart. **(B)** Schematics of the ultrasound stimulation setup. **(C)** Illustration of ultrasound stimulation parameters. **(D)** Acoustic pressure distribution of the lateral and axial direction without skull. LPS, lipopolysaccharide; i.p, intraperitoneal injection; OFT, open field test; FST, forced swimming test; TST, tail suspension test; EPM, elevated plus maze; PFC, prefrontal cortex; SD, stimulation duration; ISI, inter-stimulus interval; f, fundamental frequency; PRF, pulse repetition frequency; TBD, tone-burst duration.

After 3 days of adaptation, 1 mg/kg of lipopolysaccharide (LPS) (055:B5; Sigma-Aldrich, USA) or 0.9% saline solution was injected intraperitoneally to the experimental and the control group, respectively. Six hours after injection, mice were subjected to the OFT to exclude LPS-injected animals that did not develop sickness-like behaviors. Ultrasound intervention was then performed under isoflurane anesthesia. All behavioral tests were performed 24 h after LPS injection, and mice were then sacrificed to collect brain tissues and blood serum for biochemical testing. The experimental design is presented in Figure 1A.

### Ultrasound Apparatus and Delivery

Six hours after the LPS injection, US, or sham treatment with the power amplifier turned off were delivered for 30 min to the PFC of anesthetized mice. And the location of US was bregma anteroposterior +2.46 mm, medial-lateral 0 mm, determined with stereotaxic apparatus.

The ultrasound setup is shown in Figure 1B. Ultrasound pulse signals were generated by a signal generator (DG4162, RIGOL, Beijing, China). Pulse signals were amplified through a power amplifier (LZY-22+, MiniCircuits, Brooklyn, United States), and then transmitted to an ultrasound transducer (1905055, ndtXducer, Northborough, MA, United States) with 0.5 MHz fundamental frequency. The ultrasound transducer was connected to the head of a mouse with a conical collimator filled with degassed water. As shown in the Figure 1C, US was delivered with 100 Hz pulse repetition frequency, 50% duty cycle, 60 s stimulation duration, and 5 ms tone-burst duration. The two-dimensional sound field distribution was measured using a calibrated needle-type hydrophone (2010, Precision Acoustics, Dorchester, UK). Acoustic field distribution is shown in Figure 1D. Peak negative acoustic pressure was 0.62 MPa, and the corresponding spatial peak pulse-average intensity (I_sppa_) was 10.09 W/cm^2^.

### Behavioral Testing

#### Open Field Test

Each mouse was placed in the middle of the bottom of an open field box with size 50 × 50 × 50 cm and was allowed to move freely. A camera was fixed above the box to record for 6 min. After each test, the box was wiped with 75% alcohol to avoid the interference of excrements with the movements of the following mouse. The last 5 min videos were analyzed with Panlab (Harvard Apparatus, Holliston, MA, United States) to assess locomotor activity and emotional state.

#### Forced Swimming Test

The FST was used to assess depressive-like behaviors. Each mouse was slowly placed in a cylindrical bucket of 39 cm in height and 12 cm in diameter. The bucket was filled with 15 cm of water at a temperature of 26 ± 1°C. Each mouse remained in the water for 6 min and recorded with a camera. Mouse was removed from the water after the test, and dried with bibulous paper. Immobility time was calculated during the last 5 min of the 6 min videos.

#### Tail Suspension Test

The TST was used to evaluate depressive-like behaviors. The interval between TST and FST was 1 h. The employed TST device was a three-dimensional box with a height of 72 cm, a width of 32.5 cm, and a depth of 31 cm. The 1 cm end of the tail was hooked with adhesive tape to the device, and movements were recorded for 6 min. Immobility time was calculated during the last 5 min of the 6 min videos. After each test, the inside of the box was wiped with 75% alcohol to avoid any interference between each mouse.

#### Elevated Plus Maze Test

The elevated plus maze (EPM) test was used to evaluate the anxiety-like behaviors of the mice and 1 h interval from the previous behavioral test. The open and closed arms were 30 cm in length and placed 50 cm from the ground. Each mouse was placed at the intersection of the open and the closed arms to allow for free movement. The camera was placed directly above the EPM and recorded for 6 min. EPM apparatus was cleaned with 75% alcohol after each test. The number of entries into and the time in the open arms was recorded during the last 5 min of the 6 min videos, and videos were analyzed with Panlab (Harvard Apparatus, Holliston, MA, United States).

### Hematoxylin and Eosin Staining

To evaluate the biological safety of US, hematoxylin and eosin and Nissl staining were used to assess tissue damage. After US, the mice were anesthetized by intraperitoneal injection of chloral hydrate and were infused with saline solution and 4% paraformaldehyde. Brains were removed and placed in paraformaldehyde to be fixed overnight. The brains were then immersed in 30% sucrose at 4°C for 1–2 days. The fixed brain tissue was cut into 5 μm thick coronal slices with a microtome (CM1950; LEICA Biosystems, Wetzlar, Germany) and fixed on slides for hematoxylin and eosin staining. Briefly, each slice was stained in hematoxylin solution for 5 min, rinsed with running water for 15 min, placed into 1% hydrochloric and acetic acid for 1–3 s, and stained in eosin solution for 30 s. Each slice was further dehydrated with gradient ethanol and cleared with xylene. A panoramic scanner (Pannoramic MIDI, 3D HISTECH, Budapest, Hungary) was utilized to observe and acquire pictures of the processed sections.

### Nissl Staining

The paraffin-fixed sections were backed at 65°C for 60 min and washed three times with distilled water for 15 min. Section staining was performed using 1% toluidine blue for 10 min at 50°C. Sections were then washed with double-distilled water, and successively dehydrated in 70, 85, 95, and 100% ethanol. Finally, sections were cleared with xylene, and a drop of neutral resin was added to fix the cover glasses. Slice sections were observed under a panoramic scanner.

### Western Blot Analysis

The PFC was isolated and treated with radioimmunoprecipitation assay buffer (RIPA) (P0013B, Beyotime, China). A BCA Protein Assay Kit (P0010, Beyotime, China) was used to measure protein concentration. Equal amounts of protein were electrophoretic ally separated on a 4–20% sodium dodecyl sulfate polyacrylamide gel (P0468M, Beyotime, China). Gels were then transferred to polyvinylidene fluoride (PVDF) membranes (FFP24, Beyotime, China) with the Mini-PROTEAN Tetra System (Bio-Rad Laboratories, United States). Membranes were blocked with 5% skim milk for 1 h at room temperature and subsequently incubated overnight at 4°C with antibodies against β-actin (ab8227, 1:1,000, Abcam, United Kingdom), TNF-α (ab6671, 1:500, Abcam, United Kingdom), IL-6 (12912S, 1:1,000, Cell Signaling Technology, United States), and IL-1β (31202S, 1:1,000, Cell Signaling Technology, United States). The next day, membranes were washed three times with TBST and incubated for 1 h at room temperature with horseradish peroxidase-conjugated secondary antibodies (ab6721, 1:2,000, Abcam, United Kingdom). Proteins were detected with the BeyoECL reagent (P0018FS, Beyotime, China) through imaging system (Amercham Imager 600, Thermo Fisher Scientific, United States) after washing the membranes three times with TBST. Protein blots were analyzed with ImageJ (ImageJ, Rasband, WS, United States).

### Enzyme-Linked Immunosorbent Assay

Blood was collected at 24 h and left at room temperature until it stratified. Samples were then centrifuged at 4°C and 3,000 rpm for 4 min to extract the serum. To determine the serum levels of IL-6, IL-1β, and TNF-α, an enzyme-linked immunosorbent assay (ELISA) was performed with the MILLIPEX MAP Mouse High Sensitivity T Cell Panel (MHSTCMAG-70K, Merck, Kenilworth, NJ, United States) according to the manufacture’s protocol. To determine absorbance, the spectrophotometric value of the microplate reader was set to 450 nm, and concentration was calculated according to the standard curve.

### Immunofluorescence

Brain sections were incubated with 0.01 M PBS in 0.5% Triton X-100 (Sigma-Aldrich) and 5% bovine serum albumin (FA016, Genview), and then blocked overnight with c-Fos primary antibodies (ab208942, 1:1,000, Abcam, United Kingdom), followed by fluorescent-conjugated secondary antibodies (A0460, 1:500; Beyotime, China) for 2 h. Sections were stained at room temperature with 4,6-diamidino-2-phenylindole dihydrochloride (S2110, Solarbio Life Science & Technology, China). Images of the PFC were acquired with a panoramic scanner and analyzed with ImageJ.

### Statistical Methods

All the data were presented as mean ± standard error of the mean (SEM). SPSS v26.0 (IBM Corp., Armonk, NY, United States) was utilized for all statistical analyses. GraphPad Prism v7.0 was employed for all graphic presentations (GraphPad, La Jolla, CA, United States). To test whether inflammatory factors in the PFC could interfere with US-mediated effects on behavior, correlation analyses were conducted with OriginLab (OriginaLab, Northampton, MA, United States). The Shapiro-Wilk test was used to analyze the distribution of the data. The student’s *t*-test and one-way analysis of variance (ANOVA) were used to analyze the differences in normally distributed data. The Kruskal–Wallis test was employed for the analysis of non-normally distributed data. *P*-values (*p*) < 0.05 were considered statistically significant.

## Results

### Administration of 1 mg/kg Lipopolysaccharide Induced Pronounced Depressive-Like Behaviors

**FIGURE 2:**
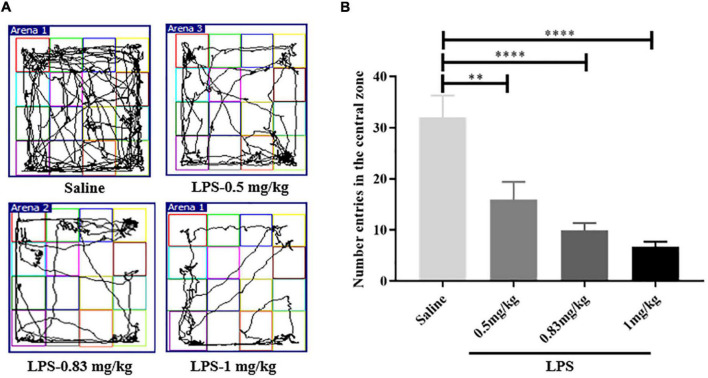
The determination of LPS experimental dose. **(A)** Motion trail of mice injected with saline and different dose of LPS. **(B)** Compared with mice injected saline, the number of entries in the central zone of LPS-injected mice was decreased, indicating depressive-like behaviors (*n* = 9 in each group). The depressive-like behaviors of mice injected with 1 mg/kg LPS was more obvious. LPS, lipopolysaccharide. **p < 0.01; ****p < 0.0001.

To determine the LPS dose used in the present study, we first performed a dose screening of LPS through open field test (OFT) (These are the doses that are commonly used in studies). As shown in Figure 2B, compared with the control group (32 ± 4.291, *n* = 9), mice injected with 0.5 mg/kg (15.89 ± 3.486, *n* = 9, *p* < 0.01), 0.83 mg/kg (9.889 ± 1.457, *n* = 9, *p* < 0.0001) and 1 mg/kg (6.667 ± 1.054, *n* = 9, *p* < 0.0001) of LPS displayed a reduction in the number entered the central zone. However, the depressive-like behaviors caused by 1 mg/kg LPS was more obvious (Figure 2). Therefore, a dose of 1 mg/kg was selected as the optimal dose for depressive-like behaviors induction in this study.

### Ultrasound Stimulation Activated c-Fos in the Prefrontal Cortex

**FIGURE 3:**
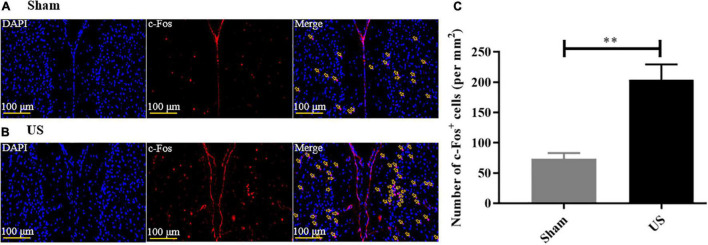
US treatment increases the expression of c-Fos in the PFC of healthy mice. Representative images of c-Fos in the PFC after sham US **(A)** and US **(B)** for 30 min (nuclei: blue; c-Fos: red). **(C)** The amount of c-Fos protein in the US-treated group (*n* = 6) is significantly greater than that in the sham group (*n* = 4). US, ultrasound stimulation; PFC, prefrontal cortex; LPS, lipopolysaccharide. **p < 0.01.

To assess the effects of US on brain activity, we measured the expression of c-Fos in the PFC of healthy mice. Immunofluorescence staining showed that US increased the excitability of PFC neurons (Figures 3A,B, and the expression of c-Fos in US-treated mice (204.00 ± 25.57, *n* = 6) was markedly increased compared to sham-treated animals (73.50 ± 9.40, *n* = 4; *p* < 0.01, Figure 3C), which indicated that US was effective in activating PFC neurons.

### Mice Developed Sickness-Like Behaviors 6 h After Lipopolysaccharide Injection

**FIGURE 4:**
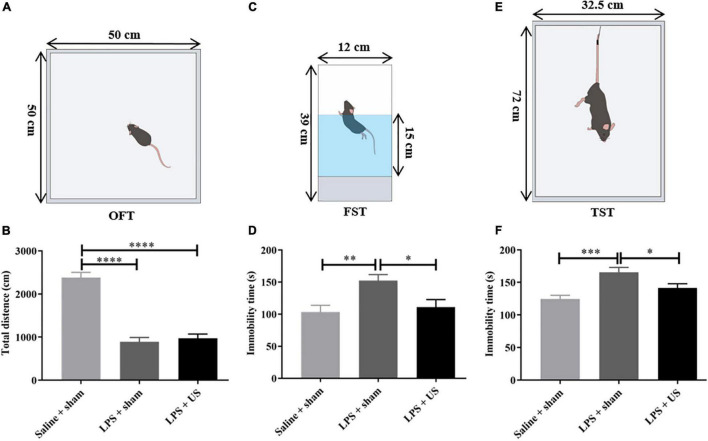
Effect of LPS and US on the behavior of healthy and depressed mice. **(A)** Schematic diagram of the OFT. **(B)** The total distance traveled in 5 min is significantly decreased in LPS-treated mice compared with mice injected saline. Schematic diagrams of the FST **(C)** and TST **(E)**. After US treatment, immobility time in the FST **(D)** and TST **(F)** is significantly reduced 24 h after LPS injection. LPS, lipopolysaccharide; US, ultrasound stimulation; PFC, prefrontal cortex; OFT, open field test; FST, forced swimming test; TST, tail suspension test. *p < 0.05; **p < 0.01; ***p < 0.001; ****p < 0.0001.

Mice clearly exhibited sickness-like behaviors 6 h post-LPS injection, including reductions in locomotion and social interaction (45). The OFT was used to determine the sickness-like behaviors, and this was also an indication of the successful animal model. Compared with control mice (2380 ± 121.1 cm, *n* = 12), the total distance traveled within 5 min in OFT was significantly reduced in mice injected with LPS (prior to US treatment) (LPS + sham: 890.80 ± 100.70 cm, *n* = 12, *p* < 0.0001, LPS + US: 971.20 ± 99.31 cm, *n* = 13, *p* < 0.0001, Figure 4B), indicating the sickness-like state of the mice.

### Ultrasound Stimulation Mitigated Lipopolysaccharide-Induced Depressive-Like and Anxiety-Like Behaviors

Intraperitoneal administration of LPS-induced behavioral alteration simulates the symptoms of depression. To verify whether US exerted antidepressant-like effect, we investigated the effect of US on immobility time in mice subjected to LPS with forced swimming test (FST) and tail suspension test (TST) and excluded the mice with improper manipulation. As shown in Figures 4D,F, LPS significantly increased the immobility time of mice in FST (Saline + sham: 103.20 ± 10.65 s, *n* = 11; LPS + sham: 152.40 ± 9.31 s, *n* = 12; *p* < 0.01) and TST (Saline + sham: 124.40 ± 5.81 s, *n* = 12; LPS + sham: 165.80 ± 7.24 s, *n* = 10; *p* < 0.001), which indicated that induction of depressive-like behaviors in mice. Nevertheless, the application of US significantly decreased the immobility time in the FST in comparison with LPS group (LPS + sham: 111.00 ± 11.70 s, *n* = 12; LPS + US: 152.40 ± 9.31 s, *n* = 12; *p* < 0.05), and the same in TST (LPS + sham: 165.80 ± 7.24 s, *n* = 10; LPS + US: 141.50 ± 6.39 s, *n* = 13; *p* < 0.05).

Comorbidity rates in anxiety and depression disorders are common (46), which suggests a partly overlapping etiology. Studies have shown that intraperitoneal injection of LPS induced anxiety-like behaviors (47, 48). Therefore, we also evaluated the effect of US on anxiety-like behaviors. As shown in Supplementary Figure 1, the reduction of entries in the open arms induced by LPS in the sham group (1.13 ± 0.35, *n* = 8) was significantly increased by US (2.75 ± 0.28, *n* = 12; *p* < 0.05), time in the open arms was improved after US treatment. In addition, after US, the total distance (Supplementary Figure 2A) and entries in the center zone (Supplementary Figure 2B) in OFT of normal mice showed no statistical difference when compared with sham group, and the immobility time in FST (Supplementary Figure 2C) also showed no difference.

### Ultrasound Stimulation Improved Lipopolysaccharide-Induced Depressive-Like Behaviors by Reducing Inflammatory Factors in the Prefrontal Cortex

**FIGURE 5:**
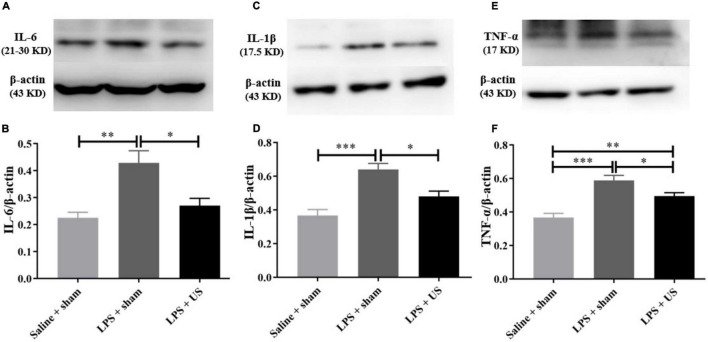
Effect of US on inflammatory factors in the PFC. **(A,C,E)** Representative Western blots of inflammatory factors in the PFC (each line represents a single animal). LPS injection increases the levels of inflammatory factors in the PFC, and US treatment decreases the expression of IL-6 **(B)**, IL-1β **(D)**, and TNF-α **(F)** in the PFC (*n* = 6 in each group). US, ultrasound stimulation; PFC, prefrontal cortex; LPS, lipopolysaccharide. *p < 0.05; **p < 0.01; ***p < 0.001.

As shown in Figure 5, the levels of inflammatory cytokines in the PFC significantly increased after LPS administration. However, US significantly decreased the LPS-induced upregulation of IL-6 (LPS + sham: 0.43 ± 0.05; LPS + US: 0.27 ± 0.03, *n* = 6 in each group; *p* < 0.05, Figure 5B), IL-1β (LPS + sham: 0.64 ± 0.04; LPS + US: 0.48 ± 0.03, *n* = 6 in each group; *p* < 0.05, Figure 5D), and TNF-α (LPS + sham: 0.59 ± 0.03; LPS + US: 0.50 ± 0.02, *n* = 6 in each group; *p* < 0.05, Figure 5F) induced by LPS. In addition, the expression of inflammation cytokines in peripheral blood serum by ELISA test showed that US did not statistically reduce the LPS induced increase in IL-6 (LPS + sham: 34.91 ± 4.05 pg/mL; LPS + US: 28.28 ± 1.46 pg/mL, *n* = 6 in each group, Supplementary Figure 4A), IL-1β (LPS + sham: 34.73 ± 6.44 pg/mL; LPS + US: 30.58 ± 2.40 pg/mL, *n* = 6 in each group, Supplementary Figure 4B) and TNF-α (LPS + sham: 2.59 ± 0.54 pg/mL; LPS + US: 2.67 ± 0.46 pg/mL, *n* = 6 in each group, Supplementary Figure 4C) in peripheral blood serum. Meanwhile, we conducted an experiment to stimulate the PFC of normal mice with US, all the procedures were same as the previous experiment design, but all the mice were normal without LPS or saline injection (*n* = 4 in each group). We found that IL-6 (Supplementary Figure 3D), IL-1β (Supplementary Figure 3E) and TNF-α (Supplementary Figure 3F) in the PFC of mice in US group shown no significant change when compared with sham group after US.

**FIGURE 6:**
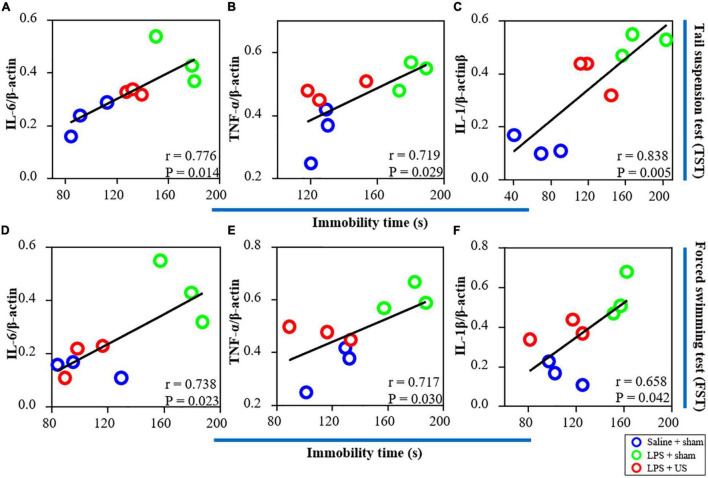
Correlations between inflammatory factors in the PFC (the results from western blot) and behavioral performance. **(A)** There is a positive correlation between the expression of IL-6 in the PFC and immobility time in the TST. The expression of TNF-α **(B)** and IL-1β **(C)** is positively correlated with immobility time in the TST. **(D)** There is a significant correlation between the expression of IL-6 in the PFC and immobility time in the FST. The expression of TNF-α **(E)** and IL-1β **(F)** is positively correlated with immobility time in the FST (*n* = 3 in each group). PFC, prefrontal cortex; US, ultrasound stimulation; LPS, lipopolysaccharide; FST, forced swimming test; TST, tail suspension test.

Furthermore, as shown in Figure 6, there was a significant correlation between the expression of IL-6 (r = 0.776, *p* = 0.014, *n* = 3, Figure 6A), TNF-α (r = 0.719, *p* = 0.029, *n* = 3, Figure 6B), and IL-1β (r = 0.838, *p* = 0.005, *n* = 3, Figure 6C) in the PFC and immobility time in the TST. In addition, a positive correlation was found between the expression of IL-6 (*r* = 0.738, *p* = 0.023, *n* = 3, Figure 6D), TNF-α (*r* = 0.717, *p* = 0.030, *n* = 3, Figure 6E), and IL-1β (*r* = 0.658, *p* = 0.042, *n* = 3, Figure 6F) in the PFC and immobility time in the FST. Thus, LPS-induced inflammation in the PFC is associated with the depressive-like phenotypes, and the improvement in LPS-induced depressive-like behaviors was due to US mediated inhibition of inflammatory cytokines in the PFC.

### Biological Safety of Ultrasound Stimulation

**FIGURE 7:**
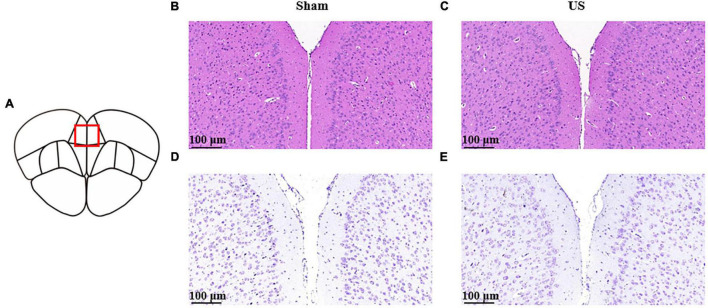
Histological analyses of PFC brain sections for the evaluation of tissue damage in sham and US-treated mice (without LPS injection). **(A)** Schematic diagram of brain sections. Representative images of hematoxylin and eosin staining in sham **(B)** and US-treated **(C)** mice. Representative images of Nissl staining in sham **(D)** and US-treated **(E)** mice (*n* = 3 in each group). PFC, prefrontal cortex; US, ultrasound stimulation; LPS, lipopolysaccharide.

To evaluate the biological safety of ultrasound used in our study, H&E staining and Nissl staining were used to analyze the tissue damage of brain area with US 30 min. As shown in Figure 7, hematoxylin and eosin staining indicated that US did not cause any damage or hemorrhage in the PFC (*n* = 3 in each group). Moreover, Nissl staining showed that the morphology and density of PFC neurons did not change after US treatment.

## Discussion

In this study, we found that US of the PFC could improve LPS-induced depressive-like and anxiety-like behaviors in mice. Compared to the sham group, US treated mice had significantly decreased levels of inflammatory cytokines in the PFC, and histological evidence showed no damage of brain tissue. Overall, these results suggested that US of the PFC may be a valid method to mitigate inflammation-induced depression.

LPS, the major component of the outer membrane of Gram-negative bacteria, is also known as a bacterial endotoxin (45). Intraperitoneal injection of LPS is a widely accepted model to mimic the depressive symptoms that occur during acute infections. Such a method has been used to screen candidate anti-depressive medications and investigate the pathophysiology of depression (49). Acute activation of the peripheral innate immune system in mice could induce depressive-like behaviors through the administration of the cytokine inducer LPS (50, 51). Inflammatory factors produced by LPS peripheral injection signal the brain to evoke central inflammation (45). The activation of powerful microglia and astrocytes in the brain and the production of pro-inflammatory cytokines accelerate neuroinflammatory responses (52), which in turn induce depressive behaviors. It has been demonstrated that systemic low-dose administration of LPS evokes time-dependent behavior alterations and manifested sickness-like and depressive-like behaviors at 6 h and 24 h post-LPS injection (53, 54). In this study, we found that intraperitoneal injection of LPS at different doses reduced the number of entries in the central zone of the OFT apparatus 24 h after LPS injection, indicating that LPS successfully induced a depressive-like state. Our findings also demonstrated that phenotypic severity is dose-dependent, as depressive-like behaviors was more obvious at a higher LPS dose, which was thus selected for our experimental induction. After US of the PFC, immobility time in the FST and TST decreased, suggesting that ultrasound significantly improves LPS-induced depressive-like behaviors. Moreover, in the EPM test, the number of US treated mice entering into and the time in the open arms increased, indicating that US of the PFC can alleviate LPS-induced anxiety-like behaviors. In addition, we also conducted an experiment to observe the effect of US of the PFC on the behavior of normal mice (without LPS or saline injection) and the expression of inflammation cytokines in PFC. However, in the OFT, there was no statistical difference in total distance traveled or number of entries in the center zone between the sham and US-treated groups (Supplementary Figures 2A,B); in addition, in the FST, immobility time remained unaltered after US treatment (Supplementary Figure 2C). Similarly, US of PFC did not affect the expression of IL-6 (Supplementary Figure 3D), IL-Iβ (Supplementary Figure 3E) and TNF-α (Supplementary Figure 3F) in PFC in normal mice. Taking together, these results suggest that US may be successfully employed for the treatment of depression.

A previous study has reported that, in a rat model subjected to 48 h restraint stress, transcranial US of the left prelimbic cortex significantly improved exploratory behavior and anhedonia. Furthermore, BDNF was markedly increased in the hippocampus (38). Another study reported that 4-week US of the PFC significantly increased sucrose intake and decreased forced swimming time in rats subjected to chronic unpredictable stress (39). Zhang et al. established a chronic unpredictable stress (CUS) model of depression in rats (39). CUS is a widely used animal model for stress induction to simulate stress encountered in life events (55, 56). Their study found that the potential mechanism of US to improve the depressive-like behaviors of CUS rats was the enhancement of BDNF/ERK/mTORC1 signaling pathway (39). However, many reports have shown that patients with depression have higher levels of inflammatory immune activation (57, 58). And the immune system signals to the brain through inflammatory cytokines and interactions between inflammation and the brain seem to promote the progression of depression (57, 59, 60). Anti-inflammatory treatments have been shown to improve depressive-like symptoms in humans and animals (61, 62). Specifically, TMS and electroconvulsive therapy have been reported to elicit antidepressant effects by attenuating the levels of inflammatory factors and downregulating immune activation (63–65). Therefore, this study considered the effect of inflammation on depression, and investigated the role of US in depressive behaviors induced by lipopolysaccharide (LPS). Our results indicated that LPS injected mice showed a significant increase in depressive-like behaviors (Figure 4) and inflammatory cytokines expression (Figure 5) in the PFC. However, 30 min of US in the PFC successfully reversed depressive-like phenotypes (Figure 4) and inflammatory activation (Figure 5). According to the results of correlation between expression of inflammation factors and depressive-like behaviors (Figure 6), we further hypothesized potential mechanisms underlying this behavioral effect could be related to the reduction of inflammation activation in the PFC. Our results provide new insights for the treatment of depression with US from the aspect of inflammatory response, and offer new evidence for the treatment of depression by ultrasound.

Higher levels of TNF-α and IL-6 have been found in the blood of patients with depression (66). This peripheral inflammation, *via* interactions with the central immune system, can trigger inflammatory responses in the brain (67). This has been demonstrated in postmortem brain tissue of patients with depression (68). Consistent with previous studies, we also observed that LPS markedly increased the expression of TNF-α, IL-Iβ, and IL-6 in the PFC and peripheral serum, inducing depressive-like and anxiety-like behaviors. US treatment significantly decreased the expression of TNF-α, IL-Iβ, and IL-6 in the PFC, and decreased serum levels of IL-6 and IL-1β, but there were no statistical differences between the sham and US groups (Supplementary Figure 2). This may indicate that the decrease in cytokine activity in the PFC may not be sufficient to result in an equivalent change in the blood serum. The reason for this could be that, in our study, US treated mice were sacrificed within a relatively short period, which might have prevented the initiation of any change in the serum levels of cytokines. Prolonged observations will be needed in further studies.

The present study has some limitations. Firstly, we established a mouse model of acute depressive-like behaviors through a single intraperitoneal injection of LPS, which may not be able to completely replicate the inflammatory activation of clinically depressed patients. Therefore, chronic inflammation in depression induced by multiple intraperitoneal injection of LPS with smaller doses should be applied in the antidepressant effects of ultrasound in subsequent studies. Secondly, the acoustic parameters used in this study are based on the work of Chang et al. (69). They demonstrated that 15 min of US at fundamental frequency of 1 MHz, pulse repetition frequency of 100 Hz, burst length of 5 ms and duty cycle of 50% attenuated the proinflammatory responses in LPS injected microglia cells. Even though we extended the stimulation to 30 min, it is possible that longer US therapy could achieve better efficacy, as 4 weeks US in the research of Zhang et al. (39). However, our depressive-like animal model was induced by a single injection of LPS, a single 30-min US may also have positive effects on model animals used in this study. Moreover, it has been shown that 15 min of US can effectively inhibit LPS-induced neuroinflammation in microglia (69), even shorter duration (10 min) of US can modulate the sensorimotor circle to promote improved neurobehavioral outcome in middle cerebral artery occlusion mice (70). In addition, the acoustic parameters used in this study are different from those of Zhang et al. (39) due to different model, which suggests that acoustic parameters should be selected for different animal models. In the following studies, we will further explore the relationship between the acoustic parameters and therapeutic effect for depression. Thirdly, while the PFC is the most common targeted brain area by brain stimulation technologies for the treatment of depression, many other deep brain regions are affected by depression, such as the hippocampus and nucleus accumbens, with a close connection between various regions. Therefore, high-resolution, deep-penetrating ultrasound should be employed in subsequent studies by using other ultrasound transducers with different focal lengths and spots to analyze such areas. Finally, we have not addressed the underlying mechanisms through which US positively affects depressive phenotypes. However, our previous study has indicated that acoustic radiation force may be involved (71). In addition to IL-6, IL-1β and TNF-α, microglia and astrocytes and their downstream molecular pathways of deep mechanism of US to alleviate inflammation need to be explored in the future.

In this study, we reported that US of the PFC can improve depressive-like behaviors in an LPS-induced murine models of depression through the reduction of brain inflammation factors. Further, we demonstrated that US is a safe procedure that does not produce damage and hemorrhage. Therefore, US is a promising tool for treatment of neuropsychological diseases. The effect of US on inflammation is worthy of further investigation to elucidate the exact mechanisms through ultrasound exerts positive effects on depression.

## Data Availability Statement

The original contributions presented in the study are included in the article/Supplementary Material, further inquiries can be directed to the corresponding authors.

## Ethics Statement

The study protocol was approved by the Animal Experimental Ethics Committee of the Shenzhen Institutes of Advanced Technology, Chinese Academy of Sciences. All animal experiments were conducted in accordance with the guidelines of Care and Use of Research Animals established by the Animal Research Committee at Shenzhen Institutes of Advanced Technology, Chinese Academy of Sciences (approval number: SIAT-IRB-150213-YGS-ZHR-A0094-2).

## Author Contributions

S-SY, LM, L-JA, X-QH, and L-LN designed the experiments and were responsible for the article writing. J-JZ, H-MC, Z-QH, X-FL, Z-RL, WZ, and S-SY performed pertinent experiment. UF and M-XC analyzed the experiment data and picture production. All authors contributed to the article and approved the submitted version.

## Conflict of Interest

The authors declare that the research was conducted in the absence of any commercial or financial relationships that could be construed as a potential conflict of interest.

## Publisher’s Note

All claims expressed in this article are solely those of the authors and do not necessarily represent those of their affiliated organizations, or those of the publisher, the editors and the reviewers. Any product that may be evaluated in this article, or claim that may be made by its manufacturer, is not guaranteed or endorsed by the publisher.

## References

[1] Kim HS Moore MT. Symptoms of depression and the discrepancy between implicit and explicit self-esteem. *J Behav Ther Exp Psychiatry.* (2019) 63:1–5. 10.1016/j.jbtep.2018.12.001 30530301

[2] Ramaker MJ Dulawa SC. Identifying fast-onset antidepressants using rodent models. *Mol Psychiatry.* (2017) 22:656–65. 10.1038/mp.2017.36 28322276

[3] Smith K. Mental health: a world of depression. *Nature.* (2014) 515:181. 10.1038/515180a 25391942

[4] Klonsky ED May AM Saffer BY. Suicide, suicide attempts, and suicidal ideation. *Annu Rev Clin Psychol.* (2016) 12:307–30. 26772209 10.1146/annurev-clinpsy-021815-093204

[5] Malhi GS Mann JJ. Depression. *Lancet.* (2018) 392:2299–312. 30396512 10.1016/S0140-6736(18)31948-2

[6] Campbell LC Clauw DJ Keefe FJ. Persistent pain and depression: a biopsychosocial perspective. *Biol Psychiatry.* (2003) 54:399–409. 10.1016/s0006-3223(03)00545-6 12893114

[7] Villa RF Ferrari F Moretti A. Post-stroke depression: mechanisms and pharmacological treatment. *Pharmacol Ther.* (2018) 184:131–44. 10.1016/j.pharmthera.2017.11.005 29128343

[8] Chi S Yu JT Tan MS Tan L. Depression in Alzheimer’s disease: epidemiology, mechanisms, and management. *J Alzheimers Dis.* (2014) 42:739–55. 10.3233/JAD-140324 24946876

[9] Schapira AHV Chaudhuri KR Jenner P. Non-motor features of Parkinson disease. *Nat Rev Neurosci.* (2017) 18:435–50. 10.1038/nrn.2017.62 28592904

[10] Gong Q He Y. Depression, neuroimaging and connectomics: a selective overview. *Biol Psychiatry.* (2015) 77:223–35. 10.1016/j.biopsych.2014.08.009 25444171

[11] Gray JP Muller VI Eickhoff SB Fox PT. Multimodal abnormalities of brain structure and function in major depressive disorder: a meta-analysis of neuroimaging studies. *Am J Psychiatry.* (2020) 177:422–34. 10.1176/appi.ajp.2019.19050560 32098488 PMC7294300

[12] Williams LM. Precision psychiatry: a neural circuit taxonomy for depression and anxiety. *Lancet Psychiatry.* (2016) 3:472–80. 10.1016/S2215-0366(15)00579-9 27150382 PMC4922884

[13] Fox ME Lobo MK. The molecular and cellular mechanisms of depression: a focus on reward circuitry. *Mol Psychiatry.* (2019) 24:1798–815. 10.1038/s41380-019-0415-3 30967681 PMC6785351

[14] Rajkowska G Miguel-Hidalgo JJ Wei J Dilley G Pittman SD Meltzer HY Morphometric evidence for neuronal and glial prefrontal cell pathology in major depression. *Biol Psychiatry.* (1999) 45:1085–98. 10.1016/s0006-3223(99)00041-4 10331101

[15] Sheline YI Wang PW Gado MH Csernansky JG Vannier MW. Hippocampal atrophy in recurrent major depression. *Proc Natl Acad Sci U S A.* (1996) 93:3908–13. 10.1073/pnas.93.9.3908 8632988 PMC39458

[16] Stockmeier CA Mahajan GJ Konick LC Overholser JC Jurjus GJ Meltzer HY Cellular changes in the postmortem hippocampus in major depression. *Biol Psychiatry.* (2004) 56:640–50. 10.1016/j.biopsych.2004.08.022 15522247 PMC2929806

[17] Drevets WC Price JL Simpson JR Jr. Todd RD Reich T Vannier M Subgenual prefrontal cortex abnormalities in mood disorders. *Nature.* (1997) 386:824–7. 10.1038/386824a0 9126739

[18] Hajszan T Dow A Warner-Schmidt JL Szigeti-Buck K Sallam NL Parducz A Remodeling of hippocampal spine synapses in the rat learned helplessness model of depression. *Biol Psychiatry.* (2009) 65:392–400. 10.1016/j.biopsych.2008.09.031 19006787 PMC2663388

[19] Morales-Medina JC Juarez I Venancio-Garcia E Cabrera SN Menard C Yu W Impaired structural hippocampal plasticity is associated with emotional and memory deficits in the olfactory bulbectomized rat. *Neuroscience.* (2013) 236:233–43. 10.1016/j.neuroscience.2013.01.037 23357118

[20] Papp M Gruca P Lason M Tota-Glowczyk K Niemczyk M Litwa E Rapid antidepressant effects of deep brain stimulation of the pre-frontal cortex in an animal model of treatment-resistant depression. *J Psychopharmacol.* (2018) 32:1133–40. 10.1177/0269881118791737 30182787

[21] Lee CW Wu HF Chu MC Chung YJ Mao WC Li CT Mechanism of intermittent theta-burst stimulation in synaptic pathology in the prefrontal cortex in an antidepressant-resistant depression rat model. *Cereb Cortex.* (2021) 31:575–90. 10.1093/cercor/bhaa244 32901273

[22] Zavorotnyy M Zollner R Rekate H Dietsche P Bopp M Sommer J Intermittent theta-burst stimulation moderates interaction between increment of N-Acetyl-Aspartate in anterior cingulate and improvement of unipolar depression. *Brain Stimul.* (2020) 13:943–52. 10.1016/j.brs.2020.03.015 32380445

[23] Eshel N Keller CJ Wu W Jiang J Mills-Finnerty C Huemer J Global connectivity and local excitability changes underlie antidepressant effects of repetitive transcranial magnetic stimulation. *Neuropsychopharmacology.* (2020) 45:1018–25. 10.1038/s41386-020-0633-z 32053828 PMC7162876

[24] Fuchikami M Thomas A Liu R Wohleb ES Land BB DiLeone RJ Optogenetic stimulation of infralimbic PFC reproduces ketamine’s rapid and sustained antidepressant actions. *Proc Natl Acad Sci U S A.* (2015) 112:8106–11. 10.1073/pnas.1414728112 26056286 PMC4491758

[25] Bystritsky A Korb AS Douglas PK Cohen MS Melega WP Mulgaonkar AP A review of low-intensity focused ultrasound pulsation. *Brain Stimul.* (2011) 4:125–36. 10.1016/j.brs.2011.03.007 21777872

[26] Kubanek J Shukla P Das A Baccus SA Goodman MB. Ultrasound elicits behavioral responses through mechanical effects on neurons and ion channels in a simple nervous system. *J Neurosci.* (2018) 38:3081–91. 10.1523/JNEUROSCI.1458-17.2018 29463641 PMC5864152

[27] Tyler WJ Tufail Y Finsterwald M Tauchmann ML Olson EJ Majestic C. Remote excitation of neuronal circuits using low-intensity, low-frequency ultrasound. *PLoS One.* (2008) 3:e3511. 10.1371/journal.pone.0003511 18958151 PMC2568804

[28] Tramontin NDS Silveira PCL Tietbohl LTW Pereira BDC Simon K Muller AP. Effects of low-intensity transcranial pulsed ultrasound treatment in a model of Alzheimer’s disease. *Ultrasound Med Biol.* (2021) 47:2646–56. 10.1016/j.ultrasmedbio.2021.05.007 34130881

[29] Beisteiner R Matt E Fan C Baldysiak H Schonfeld M Philippi Novak T Transcranial pulse stimulation with ultrasound in Alzheimer’s Disease-A new navigated focal brain therapy. *Adv Sci (Weinh).* (2020) 7:1902583. 10.1002/advs.201902583 32042569 PMC7001626

[30] Zhou H Niu L Meng L Lin Z Zou J Xia X Noninvasive ultrasound deep brain stimulation for the treatment of Parkinson’s disease model mouse. *Research (Wash D C).* (2019) 2019:1748489. 10.34133/2019/1748489 31549045 PMC6750068

[31] Zou J Yi S Niu L Zhou H Lin Z Wang Y Neuroprotective effect of ultrasound neuromodulation on kainic Acid- Induced epilepsy in mice. *IEEE Trans Ultrason Ferroelectr Freq Control.* (2021) 68:3006–16. 10.1109/TUFFC.2021.3079628 33979280

[32] Lin Z Meng L Zou J Zhou W Huang X Xue S Non-invasive ultrasonic neuromodulation of neuronal excitability for treatment of epilepsy. *Theranostics.* (2020) 10:5514–26. 10.7150/thno.40520 32373225 PMC7196311

[33] Monti MM Schnakers C Korb AS Bystritsky A Vespa PM. Non-Invasive ultrasonic thalamic stimulation in disorders of consciousness after severe brain injury: a first-in-man report. *Brain Stimul.* (2016) 9:940–1. 10.1016/j.brs.2016.07.008 27567470

[34] Bian T Meng W Qiu M Zhong Z Lin Z Zou J Noninvasive ultrasound stimulation of ventral tegmental area induces reanimation from general anaesthesia in mice. *Research (Wash D C).* (2021) 2021:2674692. 10.34133/2021/2674692 33954291 PMC8059556

[35] Cain JA Spivak NM Coetzee JP Crone JS Johnson MA Lutkenhoff ES Ultrasonic thalamic stimulation in chronic disorders of consciousness. *Brain Stimul.* (2021) 14:301–3. 10.1016/j.brs.2021.01.008 33465497

[36] Baek H Sariev A Lee S Dong SY Royer S Kim H. Deep cerebellar low-intensity focused ultrasound stimulation restores interhemispheric balance after ischemic stroke in mice. *IEEE Trans Neural Syst Rehabil Eng.* (2020) 28:2073–9. 10.1109/TNSRE.2020.3002207 32746292

[37] Liu L Du J Zheng T Hu S Dong Y Du D Protective effect of low-intensity transcranial ultrasound stimulation after differing delay following an acute ischemic stroke. *Brain Res Bull.* (2019) 146:22–7. 10.1016/j.brainresbull.2018.12.004 30552999

[38] Zhang D Li H Sun J Hu W Jin W Li S Antidepressant-Like effect of low-intensity transcranial ultrasound stimulation. *IEEE Trans Biomed Eng.* (2019) 66:411–20. 10.1109/TBME.2018.2845689 29993461

[39] Zhang J Zhou H Yang J Jia J Niu L Sun Z Low-intensity pulsed ultrasound ameliorates depression-like behaviors in a rat model of chronic unpredictable stress. *CNS Neurosci Ther.* (2021) 27:233–43. 10.1111/cns.13463 33112507 PMC7816209

[40] Medina-Rodriguez EM Lowell JA Worthen RJ Syed SA Beurel E. Involvement of innate and adaptive immune systems alterations in the pathophysiology and treatment of depression. *Front Neurosci.* (2018) 12:547. 10.3389/fnins.2018.00547 30174579 PMC6107705

[41] Maes M. Evidence for an immune response in major depression: a review and hypothesis. *Prog Neuropsychopharmacol Biol Psychiatry.* (1995) 19:11–38. 10.1016/0278-5846(94)00101-m 7708925

[42] Cotero V Fan Y Tsaava T Kressel AM Hancu I Fitzgerald P Author Correction: noninvasive sub-organ ultrasound stimulation for targeted neuromodulation. *Nat Commun.* (2020) 11:1336. 10.1038/s41467-020-15011-7 32152308 PMC7062845

[43] Zachs DP Offutt SJ Graham RS Kim Y Mueller J Auger JL Noninvasive ultrasound stimulation of the spleen to treat inflammatory arthritis. *Nat Commun.* (2019) 10:951. 10.1038/s41467-019-08721-0 30862842 PMC6414603

[44] Chen TT Lan TH Yang FY. Low-Intensity pulsed ultrasound attenuates LPS-Induced neuroinflammation and memory impairment by modulation of TLR4/NF-kappaB signaling and CREB/BDNF expression. *Cereb Cortex.* (2019) 29:1430–8. 10.1093/cercor/bhy039 30873554

[45] Zhao X Cao F Liu Q Li X Xu G Liu G Behavioral, inflammatory and neurochemical disturbances in LPS and UCMS-induced mouse models of depression. *Behav Brain Res.* (2019) 364:494–502. 10.1016/j.bbr.2017.05.064 28572058

[46] Lamers F van Oppen P Comijs HC Smit JH Spinhoven P van Balkom AJ Comorbidity patterns of anxiety and depressive disorders in a large cohort study: the Netherlands Study of Depression and Anxiety (NESDA). *J Clin Psychiatry.* (2011) 72:341–8. 10.4088/JCP.10m06176blu 21294994

[47] Sun L Ma L Zhang H Cao Y Wang C Hou N Fto deficiency reduces anxiety- and depression-like behaviors in mice via alterations in gut microbiota. *Theranostics.* (2019) 9:721–33. 10.7150/thno.31562 30809304 PMC6376469

[48] Savignac HM Couch Y Stratford M Bannerman DM Tzortzis G Anthony DC Prebiotic administration normalizes lipopolysaccharide (LPS)-induced anxiety and cortical 5-HT2A receptor and IL1-beta levels in male mice. *Brain Behav Immun.* (2016) 52:120–31. 10.1016/j.bbi.2015.10.007 26476141 PMC4927692

[49] Arioz BI Tastan B Tarakcioglu E Tufekci KU Olcum M Ersoy N Melatonin attenuates LPS-Induced acute depressive-like behaviors and microglial NLRP3 inflammasome activation through the SIRT1/Nrf2 pathway. *Front Immunol.* (2019) 10:1511. 10.3389/fimmu.2019.01511 31327964 PMC6615259

[50] Walker AK Budac DP Bisulco S Lee AW Smith RA Beenders B NMDA receptor blockade by ketamine abrogates lipopolysaccharide-induced depressive-like behavior in C57BL/6J mice. *Neuropsychopharmacology.* (2013) 38:1609–16. 10.1038/npp.2013.71 23511700 PMC3717543

[51] Wang X Zhu L Hu J Guo R Ye S Liu F FGF21 Attenuated LPS-Induced depressive-like behavior via inhibiting the inflammatory pathway. *Front Pharmacol.* (2020) 11:154. 10.3389/fphar.2020.00154 32184729 PMC7058797

[52] Sekio M Seki K. Lipopolysaccharide-induced depressive-like behavior is associated with alpha(1)-adrenoceptor dependent downregulation of the membrane GluR1 subunit in the mouse medial prefrontal cortex and ventral tegmental area. *Int J Neuropsychopharmacol.* (2014) 18:yu005. 10.1093/ijnp/pyu005 25539502 PMC4368860

[53] O’Connor JC Lawson MA Andre C Moreau M Lestage J Castanon N Lipopolysaccharide-induced depressive-like behavior is mediated by indoleamine 2,3-dioxygenase activation in mice. *Mol Psychiatry.* (2009) 14:511–22. 10.1038/sj.mp.4002148 18195714 PMC2683474

[54] Frenois F Moreau M O’Connor J Lawson M Micon C Lestage J Lipopolysaccharide induces delayed FosB/DeltaFosB immunostaining within the mouse extended amygdala, hippocampus and hypothalamus, that parallel the expression of depressive-like behavior. *Psychoneuroendocrinology.* (2007) 32:516–31. 10.1016/j.psyneuen.2007.03.005 17482371 PMC1978247

[55] Willner P Muscat R Papp M. Chronic mild stress-induced anhedonia: a realistic animal model of depression. *Neurosci Biobehav Rev.* (1992) 16:525–34. 10.1016/s0149-7634(05)80194-0 1480349

[56] Antoniuk S Bijata M Ponimaskin E Wlodarczyk J. Chronic unpredictable mild stress for modeling depression in rodents: meta-analysis of model reliability. *Neurosci Biobehav Rev.* (2019) 99:101–16. 10.1016/j.neubiorev.2018.12.002 30529362

[57] Beurel E Toups M Nemeroff CB. The bidirectional relationship of depression and inflammation: double trouble. *Neuron.* (2020) 107:234–56. 10.1016/j.neuron.2020.06.002 32553197 PMC7381373

[58] Nowak W Grendas LN Sanmarco LM Estecho IG Arena AR Eberhardt N Pro-inflammatory monocyte profile in patients with major depressive disorder and suicide behaviour and how ketamine induces anti-inflammatory M2 macrophages by NMDAR and mTOR. *EBioMedicine.* (2019) 50:290–305. 10.1016/j.ebiom.2019.10.063 31753725 PMC6921226

[59] Miller AH Raison CL. The role of inflammation in depression: from evolutionary imperative to modern treatment target. *Nat Rev Immunol.* (2016) 16:22–34. 10.1038/nri.2015.5 26711676 PMC5542678

[60] Kiecolt-Glaser JK Derry HM Fagundes CP. Inflammation: depression fans the flames and feasts on the heat. *Am J Psychiatry.* (2015) 172:1075–91. 10.1176/appi.ajp.2015.15020152 26357876 PMC6511978

[61] Kappelmann N Lewis G Dantzer R Jones PB Khandaker GM. Antidepressant activity of anti-cytokine treatment: a systematic review and meta-analysis of clinical trials of chronic inflammatory conditions. *Mol Psychiatry.* (2018) 23:335–43. 10.1038/mp.2016.167 27752078 PMC5794896

[62] Tomaz VS Chaves Filho AJM Cordeiro RC Juca PM Soares MVR Barroso PN Antidepressants of different classes cause distinct behavioral and brain pro- and anti-inflammatory changes in mice submitted to an inflammatory model of depression. *J Affect Disord.* (2020) 268:188–200. 10.1016/j.jad.2020.03.022 32174477

[63] Tian L Sun SS Cui LB Wang SQ Peng ZW Tan QR Repetitive transcranial magnetic stimulation elicits antidepressant- and anxiolytic-like effect via nuclear factor-E2-related Factor 2-mediated anti-inflammation mechanism in rats. *Neuroscience.* (2020) 429:119–33. 10.1016/j.neuroscience.2019.12.025 31918011

[64] Hestad KA Tonseth S Stoen CD Ueland T Aukrust P. Raised plasma levels of tumor necrosis factor alpha in patients with depression: normalization during electroconvulsive therapy. *J ECT.* (2003) 19:183–8. 10.1097/00124509-200312000-00002 14657769

[65] Guloksuz S Rutten BP Arts B van Os J Kenis G. The immune system and electroconvulsive therapy for depression. *J ECT.* (2014) 30:132–7. 10.1097/yct.0000000000000127 24755720

[66] Dowlati Y Herrmann N Swardfager W Liu H Sham L Reim EK A meta-analysis of cytokines in major depression. *Biol Psychiatry.* (2010) 67:446–57. 10.1016/j.biopsych.2009.09.033 20015486

[67] Maier SF. Bi-directional immune-brain communication: implications for understanding stress, pain, and cognition. *Brain Behav Immun.* (2003) 17:69–85. 10.1016/s0889-1591(03)00032-1 12676570

[68] Enache D Pariante CM Mondelli V. Markers of central inflammation in major depressive disorder: a systematic review and meta-analysis of studies examining cerebrospinal fluid, positron emission tomography and post-mortem brain tissue. *Brain Behav Immun.* (2019) 81:24–40. 10.1016/j.bbi.2019.06.015 31195092

[69] Chang JW Wu MT Song WS Yang FY. Ultrasound stimulation suppresses LPS-Induced proinflammatory responses by regulating NF-kappaB and CREB activation in microglial cells. *Cereb Cortex.* (2020) 30:4597–606. 10.1093/cercor/bhaa062 32248223

[70] Deng LD Qi L Suo Q Wu SJ Mamtilahun M Shi RB Transcranial focused ultrasound stimulation reduces vasogenic edema after middle cerebral artery occlusion in mice. *Neural Regen Res.* (2022) 17:2058–63. 10.4103/1673-5374.335158 35142697 PMC8848588

[71] Ye J Tang S Meng L Li X Wen X Chen S Ultrasonic control of neural activity through activation of the mechanosensitive channel MscL. *Nano Lett.* (2018) 18:4148–55. 10.1021/acs.nanolett.8b00935 29916253

