# Supplementary material for: Ultrasound Stimulation of Prefrontal Cortex Improves Lipopolysaccharide-Induced Depressive-Like Behaviors in Mice
Source: Front Psychiatry. 2022 Apr 29;13:864481. doi: 10.3389/fpsyt.2022.864481 (PMC9099414; doi:10.3389/fpsyt.2022.864481)
Supplement: Supplementary file 1 [file Presentation_1.pdf]

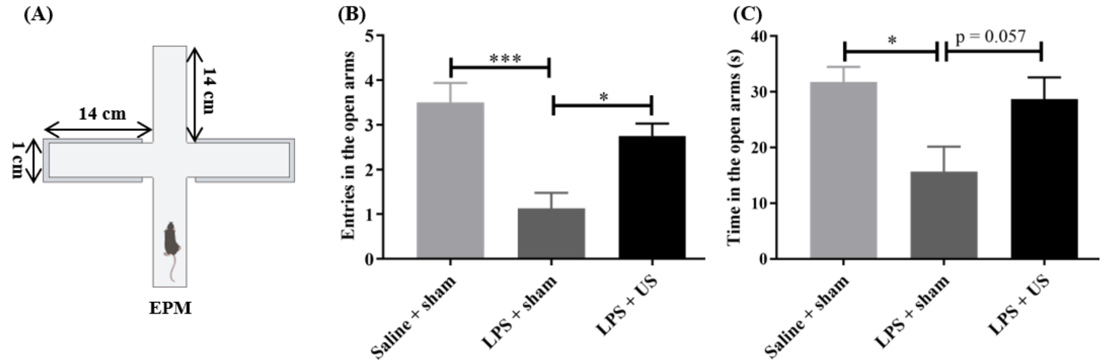

Supplementary Figure 1 (A) The schematic diagram of Elevated plus maze (EPM). (B) Entries in the open arms of the depression mice statistically increase in comparison with the mice in sham stimulation group after US. (C) With the treatment of US, the time in the open arms was increased (Saline + sham: n = 12; LPS + sham: n = 8; LPS + US: n = 12). LPS: lipopolysaccharide; US: ultrasound stimulation. ***, $p < 0.01$; *, $p < 0.05$.

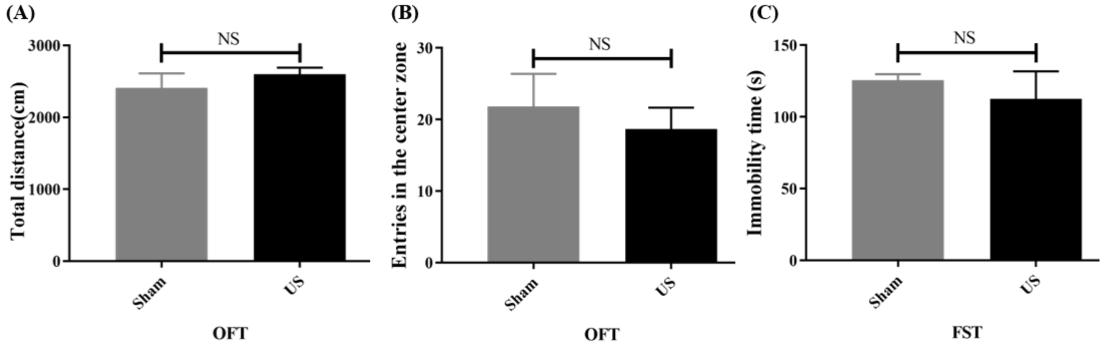

Supplementary Figure 2 The effect of ultrasound stimulation on behavioral tests of normal mice. In open field test (OFT) (n = 6 in each group), there was no significant alteration in total distance (A) and entries in the center zone (B) between sham and US group. And in forced swimming test (FST) (n = 6 in each group), the immobility time of mice with ultrasound stimulation presented no statistical decrease when compared with mice in sham group (C). US: ultrasound stimulation; NS: no significant.

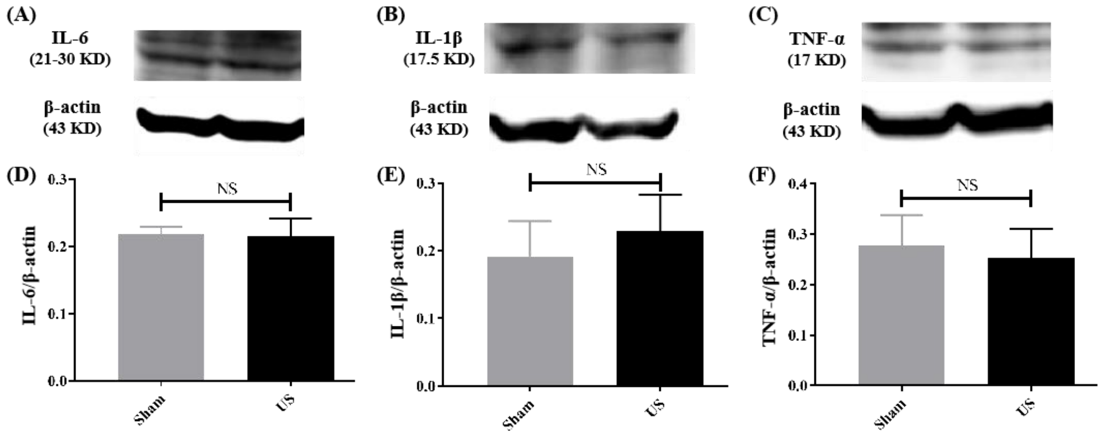

Supplementary Figure 3 The effect of ultrasound stimulation on the expression of inflammation cytokines in PFC of normal mice. (A, B, C) Representative Western blots of inflammatory factors in the PFC (each line represents a single animal). For the normal mice, ultrasound stimulation on the PFC did not alter the expression of IL-6 (D), IL-1β (E), and TNF-α (F) in PFC (n = 4 in each group). US: ultrasound stimulation; PFC: prefrontal cortex; NS: no significant.

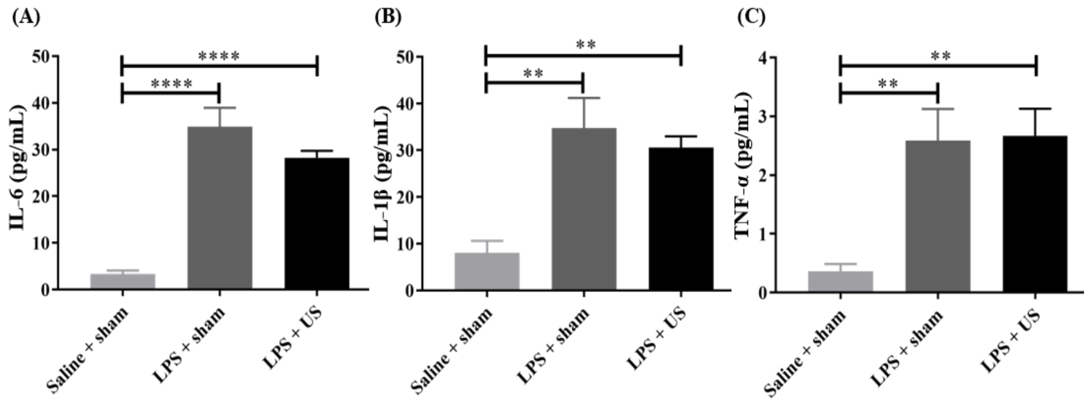

Supplementary Figure 4 The expression of inflammatory cytokines in peripheral blood serum were checked by ELISA. Compared with Saline + sham group, intraperitoneal injection of LPS resulted in significant increases of IL-6 (A) (n = 6 in each group), IL-1β (B) (n = 6 in each group) and TNF-α (C) (n = 6 in each group) in peripheral serum. However, US of PFC did not significantly reverse the increase of inflammatory cytokines in peripheral blood serum. LPS: lipopolysaccharide; US: ultrasound stimulation; PFC: prefrontal cortex. ****, $p < 0.001$; **, $p < 0.01$.
